# Preservation of global synaptic excitatory to inhibitory ratio during long postmortem intervals

**DOI:** 10.1038/s41598-020-65377-3

**Published:** 2020-05-25

**Authors:** Pietro Scaduto, Adolfo Sequeira, Marquis P. Vawter, William Bunney, Agenor Limon

**Affiliations:** 1 Department of Neurology, Mitchell Center for Neurodegenerative Diseases. School of Medicine. University of Texas Medical Branch at Galveston, Galveston, USA; 2 Department of Biomedicine, Neuroscience and Advanced Diagnostics, Division of Human Physiology, University of Palermo, 90134 Palermo, Italy; 3 Department of Psychiatry & Human Behavior, University of California Irvine, Irvine, CA 92697 USA

**Keywords:** Voltage clamp, Ion channels in the nervous system

## Abstract

The study of postsynaptic excitation to inhibition (E/I ratio) imbalances in human brain diseases, is a highly relevant functional measurement poorly investigated due to postmortem degradation of synaptic receptors. We show that near-simultaneous recording of microtransplanted synaptic receptors after simulated morgue conditions allows the determination of the postsynaptic E/I ratio for at least 120 h after death, expanding the availability and use of human diseased tissue stored in brain banks.

## Introduction

The excitation to inhibition balance has been defined as the average amount of depolarizing to hyperpolarizing neuronal synaptic currents (global synaptic E/I ratio or just E/I ratio) in a particular brain region^1^. Because the E/I ratio emerges from complex processes that are critical for physiological coding by neural circuits ensembles^2^, alterations of this balance have been proposed to underlie neuropsychiatric and neurodegenerative disorders such as schizophrenia, autism, and dementia^3,4^. However, to what extent potential disturbances of the E/I ratio in brain disorders are actually corrected by synaptic scaling^5^, heterosynaptic plasticity^6^ and changes of synaptic function^7^ is not known. Because global synaptic E/I imbalances cannot be implied from observing dysfunction in only one component, simultaneous measurements of excitatory and inhibitory components are necessary to directly assess whether a putative synaptic E/I imbalance has been, or not, corrected by homeostatic processes. Thus, accurate measurement of E/I imbalances in human brain synapses is critical to understand the pathophysiology of brain disorders whose major clinical manifestations emerge from abnormal synaptic function. Whereas the E/I ratio has been measured at the neuronal level in animal models, the overall synaptic ratio in a particular region of the brain is still not well known. In humans the E/I ratio is even less understood due to the difficulty to measure the simultaneous activity of synaptic excitatory AMPA receptors (AMPARs) and inhibitory GABA_A_ receptors (GABA_A_Rs) in living people. The reactivation of postmortem tissue by microtransplantation of synaptic membranes (MSM), which allows the electrophysiological analysis of postsynaptic AMPARs and GABA_A_Rs^8–11^, has opened the possibility to determine E/I imbalances in human brains. However, the large variability of ion current responses across microtransplanted cells^12–14^ and the potential degradation of biological activity of ion channels due to postmortem interval (PMI)^15^, has slowed the study of E/I imbalances in disorders involving synaptic alterations. Our initial MSM experiments have shown preservation of synaptic GABA receptors for PMI as long as 29 h without apparent loss of function in 4 control subjects^16^. Those results prompt us to investigate the limits of the preservation of synaptic receptors, and its potential use to determine the synaptic E/I ratio. In this proof of principle study, we show that the global postsynaptic E/I ratio can be determined from the near-simultaneous recording of postmortem synaptic AMPARs and GABA_A_Rs microtransplanted into *Xenopus* oocytes, and that the large variability of responses observed when AMPARs or GABARs are studied separately is greatly minimized when the E/I ratio is used instead. We also demonstrate that the E/I ratio is relatively unaffected by PMI when the tissue is stored at cold temperatures even for long periods of time.

## Methods

### Rat brain cortex

**Figure 1:**
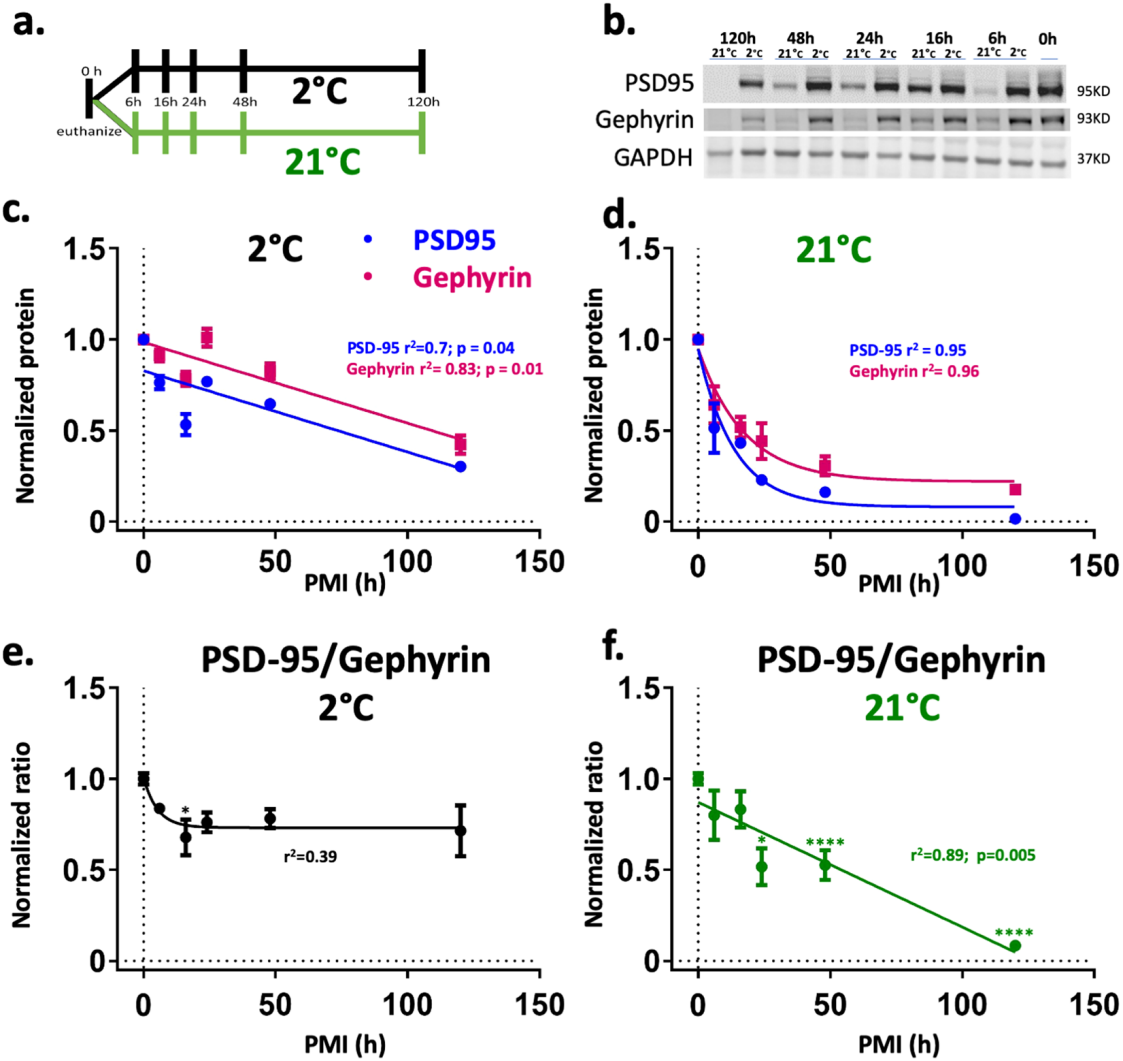
Morgue temperature reduced the rate of synaptic protein degradation and preserved the PSD-95/gephyrin ratio. (**a**) Schematic of experimental schedule. (**b**) Representative Western blots of excitatory (PSD-95), inhibitory (gephyrin) postsynaptic markers, and GAPDH that was used as an internal control in synaptosome-enriched P2 fractions. The bands were cropped from the full-length gel displayed in supplementary Fig. 4a. (**c–d**) Synaptic protein levels of PSD-95 and gephyrin at 2 °C and 21 °C. Linear regressions (2 °C) and one phase decay (21 °C) functions were used to fit the means of gephyrin and PSD-95 protein level along the PMI. (**e–f**) The PSD-95/gephyrin ratio shows temperature-dependent relationships across time. At simulated morgue conditions, and after an initial reduction, the PSD-95/gephyrin ratio is preserved at least after 120 hrs after death. The E/I ratio was fitted with one phase decay at 2 °C and with a linear regression at 21 °C. Here and in next figures the p values and r^2^ are shown for linear regresions; non-linear regressions show the r^2^. Data are reported as means ± SEM. *p  <  0.05, ***p < 0.0001. N = 8,7,5,4,4,4 gel bands for 0,6,16,24,48,120 h at 2 °C and N = 8,7,5,4,4,4 gel bands for 0,6,16,24,48,120 h at 21 °C (Supplementary Table 1). Statistical test is a One-way ANOVA followed by multiple comparison Dunnett’s test *vs* the control group (time 0).

Twenty two adult male Wistar rats, 2 months old, were euthanized at the same time following procedures in accordance with the National Institutes of Health Guide for the Care and Use of Laboratory Animals. The experimental protocols were approved by the IACUC at University of California, Irvine (IACUC: 1998–1388). According to the *Requirements for the facilities and operation of mortuaries* by the National Pathology Accreditation Advisory Council (NPAAC) the morgue temperature range for humans is 2–6 °C^17^. Therefore, euthanized rats were kept at 2 °C, to simulate morgue conditions, or 21 °C, to simulate room temperature, for different time intervals: 0, 6, 16, 24, 48, or 120 h (N = 2 rats for each experimental point; 22 rats in total). The body temperature of the rats was measured by putting a small digital temperature logger (ibutton; thermochron; Baulkham Hills, Australia) outside the chest of the animals. At the specified time brains were surgically removed; the cortex was isolated, frozen by immersion in liquid nitrogen and stored at −80 °C (Fig. 1a).

### Human tissue

The right frontal hemisphere from a 39 years old male control subject (PMI = 15 hrs) was obtained postmortem from the University of California Irvine Brain Bank (UCIBB) after obtaining verbal and written consent from next of kin according to guidelines of the Institutional Review Board approval. The UCIBB protocols for human brain tissue were approved by the Institutional Review Board at University of California, Irvine (HS#1997–74). A psychological autopsy was completed based on family informant information, medical and psychiatric records, toxicology reports, and the subject’s medication history. The UCIBB autopsy protocol is based largely on procedures validated by Kelly and Mann^18^, and includes questions concerning the decedents’ demographics, medical history, psychiatric symptoms, medication use, hospitalizations, substance use, physical health. The human brain dissection and freezing protocol is described in detail elsewhere^19^. Briefly, after collection, frontal cortex samples were either frozen in isopentane at −40 °C (PMI = 15 h) or keept at 4 °C at different time intervals (18, 21, 27, 39, 63, and 87 h) before freezing. This temperature is in the middle of the range recommended by the NPAAC. It is important to note that that due to the limited amount of human brain donations of “control” people, we limited the use of brain specimens in experiments where degradation and loss of function is expected. For this reason, we only include specimens from one human brain and most of our work was done with brain tissue from rats.

### Synaptosomes isolation

Synaptosomal enriched preparations were extracted from 50 mg of human DLPFC or 60 mg of dissected rat cortex using Syn-PER reagent protocol (Thermo Scientific, Rockford, IL) following the manufacturer instructions. We pooled brain tissue from the 2 rats used for each condition to obtain a single preparation per time point and temperature. Three fractions (S1, P1, P2) from each preparation were isolated, stored at −80 °C, and used for downstream protocols as specified below. The S1 fraction contains soluble cytosolic elements. The P2 fraction is enriched in synaptosomes. The P1 fraction, which contains mostly nuclei, myelin, and large non-homogenized tissue, was used as a reference for synaptosomes enrichment in P2 fractions by Western Blot analysis. All the procedures of centrifugation were performed at 4 °C using proteinase inhibitors (Thermo fisher, cat #A32955) to reduce proteolysis and denaturation. P1 and P2 fractions were re-suspended in Syn-Per buffer solution and the amount of protein was quantified by DeNovix QFX fluorometer instrument (DeNovix Inc. Wilmington, USA) and Qubit reagents (Invitrogen, cat# Q33212).

### Western blotting

Twenty μg of proteins from P2 fractions were run in a polyacrylamide gel (Invitrogen precast gradient gels 4–12% bis-tris plus) at 155 V. To reduce the variability multiple proteins were detected by re-blotting the membrane with different antibodies and without a stripping protocol between blottings. Proteins were transferred from the gel to a nitrocellulose membrane (Invitrogen, cat#LC2000) and unspecific blocking was reduced by incubating membranes for 1 h at room temperature with Odyssey blocking buffer (LI-COR Biosciences - U.S.). Next, membranes were treated for 1 h at room temperature, or overnight at 4 °C, with the following primary antibodies. Mouse anti-synaptophysin (Abcam cat# ab8049, dilution 1:2000), mouse anti-PSD-95 antibody (Thermo Fisher cat# MA1–045, dilution 1:2500), rabbit anti-gephyrin antibody (Abcam cat# ab32206, dilution 1:1600), rabbit anti-NeuN antibody (Abcam, cat#ab177487, rabbit, and dilution 1:2000), rabbit anti-GAPDH antibody (Abcam cat# ab9485, rabbit, and dilution 1:3000). GAPDH was selected because is minimally affected by PMI^20^. To visualize the primary antibody membranes were incubated with fluorescent secondary antibody (Li-cor anti-rabbit cat#926–32213 and anti-mouse cat#926–68072 dilution 1:2000 for both) and imaged with an Odyssey infrared imager (Li-Cor Odyssey 9120 Infrared Imaging System, Lincoln, Nebraska USA). The intensities of the bands were analyzed with Image J software (Rasband WS, ImageJ, U.S. National Institutes of Health, Bethesda, MD, http://rsb.info.nih.gov/ij/, 1997–2017). Statistical significance was evaluated by One-way ANOVA comparing the values at different time points vs the control (the lowest PMI) within the same temperature. For post hoc analysis we used Dunnett’s multiple comparison test (JMP version 14; SAS Institute, Cary, NC).

### Analysis of pH

Human and rat brain cortex (60 mg) were homogenized at 4 °C in 600 µL of ddH_2_O at 7 pH (w/v 1:10) as previously described^21^. After 30 minutes, the pH was measured using a micro pH electrode and the Accumet AE150 pH Benchtop Meter (Thermo Fisher Scientific Inc.). Three readings per sample were taken. Intraclass correlation coefficient [ICC] was calculated using R studio software, ICC = 0.999,95% confidence interval [CI] within samples.

### Isolation and injection of RNA

Total RNA was extracted from the P2 fraction from each rat experimental group using PureLink RNA Mini Kit (Invitrogen by Thermo Fisher Scientific). Extracted RNA was quantified using NanoDrop 2000/2000c and was later injected into the oocytes (2 mg/mL). Electrophysiological recordings of oocytes injected with total RNA were performed 24 h after injection. Quality of isolated RNA was determined by RIN value using an Agilent 2100 bioanalyzer.

### Microtransplantation of synaptic membranes (MSM) and TEVC

For MSM experiments 50 nL of P2 fractions at 2 µg/µL were injected into stages V-VI *Xenopus Laevis* oocytes as previously described^11^ and recorded by TEVC the day after the microinjection (20–28 h). Briefly, frogs were placed in anesthetic solution (0.17% MS-222) for 10–15 min before extracting the ovaries; following procedures in accordance with the National Institutes of Health Guide for the Care and Use of Laboratory Animals, the IACUC at the University of California, Irvine (IACUC: 1998–1388), and at the University of Texas Medical Branch at Galveston (IACUC:1803024). Oocytes were isolated and defolliculated by carefully stirring them in a solution containing 2 mg/mL collagenase for 2 h at 30 °C; then, oocytes were transferred to a Petri dish containing Barth’s solution [88 mM NaCl, 1 mM KCl, 0.41 mM CaCl2, 0.82 mM MgSO4, 2.4 mM NaHCO3, 5 mM HEPES (pH 7.4)], and placed in a temperature-controlled environment at 16 °C for 24 h as has been previously reported^12,13,22^. Stage V–VI oocytes were manually separated and placed in a fresh Barth’s solution for injection of synaptosomal enriched membranes. P2 fractions, harboring synaptic receptors, were suspended in sterile distilled water and sonicated to create small proteoliposomes that can fuse to the oocytes’ extracellular membrane^11^. For TEVC experiments microelectrodes were filled with 3 M KCl and resistance of the microelectrodes ranged from 0.5 to 3.0 MΩ. Piercing and recording took place in a chamber (volume ≈ 0.1 ml) continuously perfused (5–10 ml/min) with Ringer’s solution [115 mM NaCl, 2 mM KCl, 1.8 mM CaCl_2_, 5 mM Hepes (pH 7.4)] at room temperature (19–21 °C). Oocytes were voltage clamped to −80 mV using an Oocyte Clamp OC-725C amplifier. Ion currents were recorded and stored with WinEDR version 2.3.8 Strathclyde Electrophysiology Software (John Dempster, Glasgow, United Kingdom) as previously reported^11,13^. Currents were filtered by Dual variable filter Kemo at 20 Hz. GABA was from Sigma (St. Louis, MO, USA) and kainate from Tocris (Minneapolis, MN, USA). All the drugs were dissolved into Ringer’s solution. To estimate the E/I ratio, GABA and kainate were applied once to each microtransplanted oocyte. Data per each PMI interval are shown as the mean ± S.E.M of the oocytes tested for each condition. The number of microtransplanted oocytes tested per each condition (technical replicates) was determined by analyzing the magnitude of the effect and the dispersion of the variability as previously reported^11^. For an exact number of the oocytes tested please see supplementary Tables 1–5. Statistical differences were determined by one-way ANOVA, followed by Dunnett’s multiple comparisons test between the values at different time points *vs* the control (the lowest PMI) within the same temperature. Data was considered significant when *p*  <  0.05. (Graphpad Prism v.8). The EC_50_ for GABA and kainate was determined by fitting the Hill equation in the form I  =  100/(1  +  10^(LogEC_50_-[A])*Hill slope), in which I is the current amplitude, 100 is the maximum normalized current at the concentration of the agonist [A], EC_50_ is the agonist concentration that induces 50% of the maximal response (Graphpad Prism v.8). The experimental data are shown as the mean ± S.E.M. Statistical differences were determined by one-way ANOVA and considered significant when *p*  <  0.05 (Graphpad Prism).

## Results and Discussion

To test the PMI effects on the E/I ratio, 22 adult Wistar rats were euthanized at room temperature (21 °C). The cerebral cortex was immediately removed from 2 rats, snap-frozen in liquid nitrogen and stored at −80 °C for downstream applications. These rats were used as controls (PMI = 0 h). The remaining rats were separated into two groups. One group was kept at room temperature (21 °C) and the other was stored inside a refrigerator (2 °C) to simulate morgue conditions. Cerebral cortices from 2 rats per time point (6, 16, 24, 48 and 120 hrs), per group, were surgically removed and snap-frozen in liquid nitrogen. The effects of PMI, from 15 to 87 hrs, were also evaluated on tissue samples from the frontal pole of a 39 years old male subject with no history of drug abuse, psychiatric or neurodegenerative disorders. After euthanasia, the rats body temperature fell exponentially from 38 °C to 21 °C with a time constant (*τ*) of 7.5 hrs, and to 2 °C with a *τ* of 4.8 hrs (supplementary Fig. 1a). Measurements of pH in brain rat cortex homogenates showed that the PMI had no significant effect on pH when the rat bodies were kept at 2 °C for up to 120 hrs after death (pH = 6.80 ± 0.04; *F* (5,12) = 3.096, *p* = 0.0505; one-way ANOVA; supplementary Fig. 1b). However, a significant acidification at room temperature was observed at 48 and 120 hrs (pH = 6.74 ± 0.1; *F* (5, 12) = 22.53, *p* < 0.0001; one-way ANOVA followed by Dunnett’s multiple comparisons test). The pH of human brain specimens showed an oscillation across PMI and we found a significative difference at 18 h PMI compared to the lowest PMI at 15 h (pH = 6.69± 0.07; *F* (6,14) = 11.17, p = 0.0001 one-way ANOVA, followed by Dunnett’s multiple comparisons test, supplementary Fig. 1c). Interestingly, the pH oscillation in rat and human brain tissue stored at morgue temperatures seems to follow a similar pattern across the PMI. In rats we measured RNA integrity (RIN) values that were preserved at 2 °C for up to 120 hrs (7.4 ± 0.37), and at 21 °C for up to 48 hrs (7.3 ± 0.39) (supplementary Fig. 2a). Synaptic RNA from rats kept at room temperature was highly degraded by 120 hrs.

Degradation level and loss of biological activity of synaptic receptors in  synaptosomes were evaluated in synaptosome-enriched P2 membrane preparations. Synaptosome enrichment was determined by the ratio of synaptophysin (Syn) to the nuclear marker NeuN, measured by WB (Syn/NeuN) (supplementary Fig. 3). The Syn/NeuN ratio in the P2 fraction was increased by 603 ± 106% at 2 °C across all PMIs, and by 622 ± 278% at 21 °C for PMIs up to 48 hrs, compared to the P1 fraction. In agreement with the decline of the degradation markers pH and RIN, samples from brains left for 120 hrs at 21 °C showed no synaptosome enrichment. With exception of 21 °C/120 hrs samples, similar synaptosome enrichment across different PMIs was observed at 2 °C and 21 °C (2 °C *F* (11,26) = 4.489, *p* < 0.0008 and 21 °C *F* (11,20) = 4.423, p = 0.002, one-way ANOVA); although a loss of enrichment trend was observed at 21 °C (r = −0.916, Pearson’s correlation coefficient). WB analysis of the excitatory and inhibitory postsynaptic density proteins, PSD-95 and gephyrin, showed a time-dependent linear reduction of the detection of these proteins at 2 °C (Fig. 1). Notably, the abundance of both proteins was highly correlated across PMIs indicating a uniform degradation (Fig. 1c); consequently, the PSD-95/gephyrin ratio, at 2 °C, remained borderline constant up to 120 hrs after death *F* (5,26) = 2.419, *p* = 0.063 (Fig. 1e). At room temperature the degradation of PSD and gephyrin was better described by a one-phase decay equation (Fig. 1d) and PSD-95 was more affected compared to gephyrin leading to a significantly lower PSD-95/gephyrin ratio after 24 h at room temperature (Fig. 1f).

**Figure 2:**
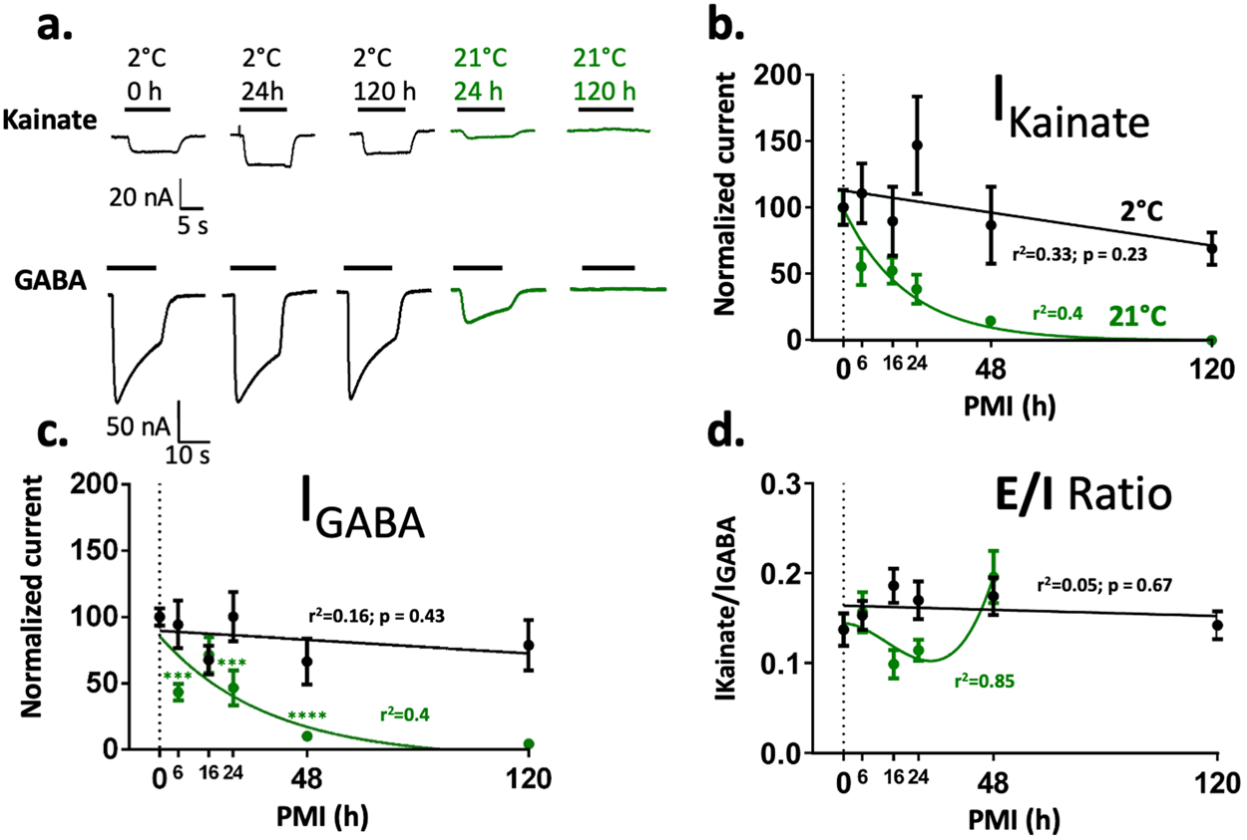
Electrophysiological activity of rat native synaptic receptors is preserved by low temperature. (**a**) Ion current responses of oocytes microtransplanted with rat synaptic receptors measured by Two-Electrodes Voltage Clamp. (**b–c**) Maximum current of synaptic receptors normalized to the control (PMI = 0). The currents generated by GABA and glutamate receptors, across PMIs time points, are well preserved at 2 °C but not at 21 °C showing significant differences compared to the control (time 0) (N = 7–11 oocytes per point, for a detailed description of the number of oocytes in each point, please see supplementary Table 2). Black and green lines represents linear regressions and one-phase decay fits, respectively. (**d**) The E/I ratio (kainate/GABA current) was calculated only in oocytes that have a clear signal for both GABA and kainate currents. Data shown is the mean  ± SEM of 7–10 oocytes per point; please see supplementary Table 1). No differences were observed across groups. Statistical analysis was done using one-way ANOVA test, followed by Dunnett’s multiple comparison test where appropriate. Statistical differences where *p < 0.05, **p < 0.01; ***p < 0.001; **** p < 0.0001). The mean values of the E/I ratio were fit with a linear regression (black) and a third order polynomial equation (green).

**Figure 3:**
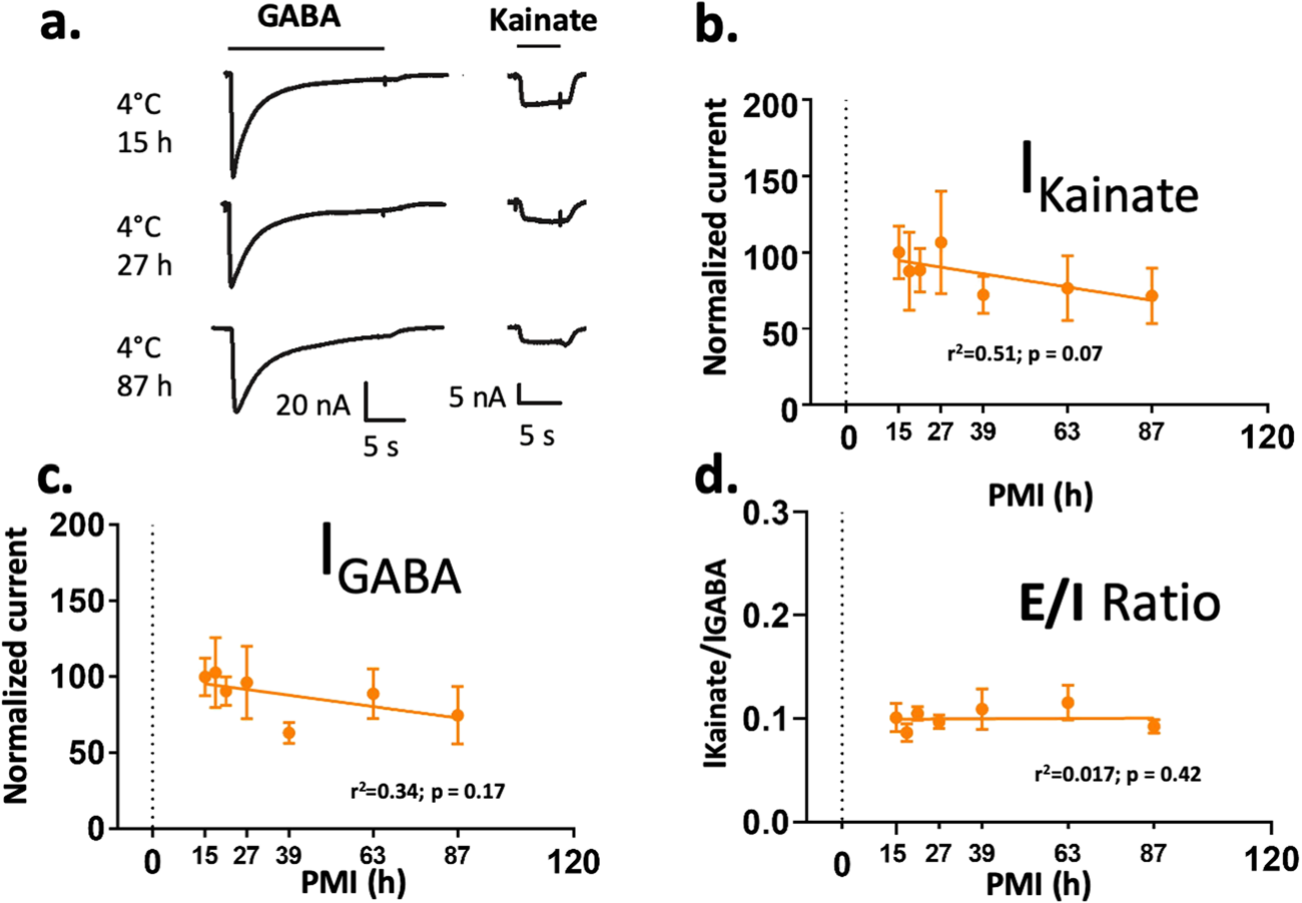
Electrophysiological activity of cortical human native synaptic receptors is preserved by low temperature. (**a**) Kainate and GABA currents recorded from microtransplanted human synaptic receptors. (**b–c**) Maximum current of synaptic receptors normalized to the control (initial PMI = 15 h). The mean values of maximal currents were fitted using a linear regression. No differences were found using One-way ANOVA test, followed by Dunnett’s multiple comparison test (N = 6–12 oocytes per point for GABA; 3–11 oocytes per point for kainate; 3–11 oocytes per point for E/I ratio, see supplementary Table 3 for details of the N per point) (**d)** E/I ratio of human synapses does not show significant change across PMI.

**Figure 4:**
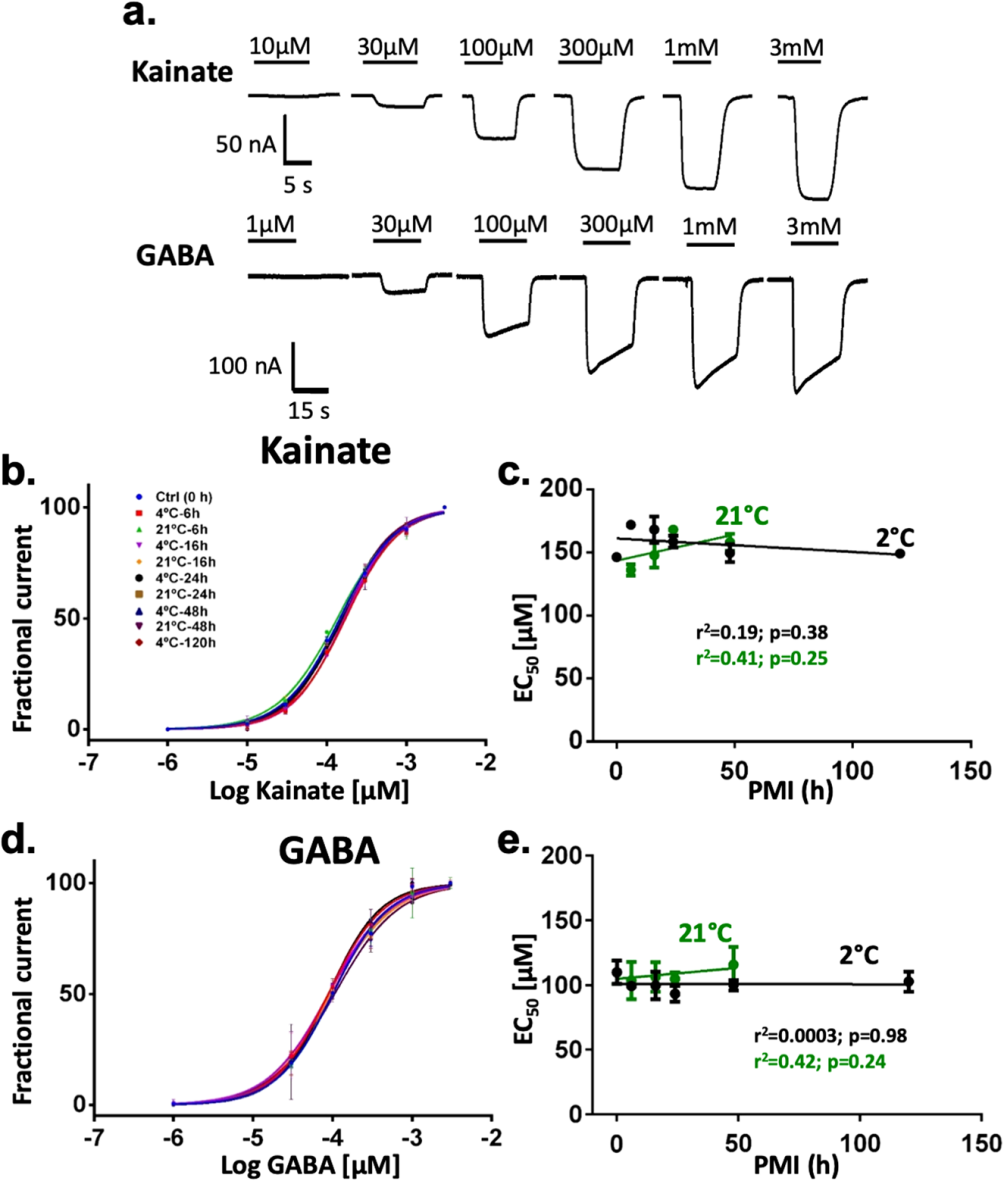
Synaptc receptor affinity is maintained for at least 5 days at 2 °C and 2 days at 21 °C. (**a**) Ion currents elicited by different concentrations of GABA and kainate in two oocytes microtransplanted with rat synaptic receptors. Only oocytes with full dose responses and fits with p values <0.05 were included in the analysis. (**b–d)** Concentration response curves for kainate and GABA (each point was calculated as the mean of 2–3 microtransplanted oocytes; see supplementary Table 4). Curve fitting was done using the Hill equation I_current_ = 100/[1 + 10^((LogEC_50_-[agonist])*HillSlope)]. (**c–e**) EC_50_ (mean + SEM) was calculated for each dose-response curve. 21 °C/120 hrs had no current responses. All the groups were fit with a linear regression equation.

**Figure 5:**
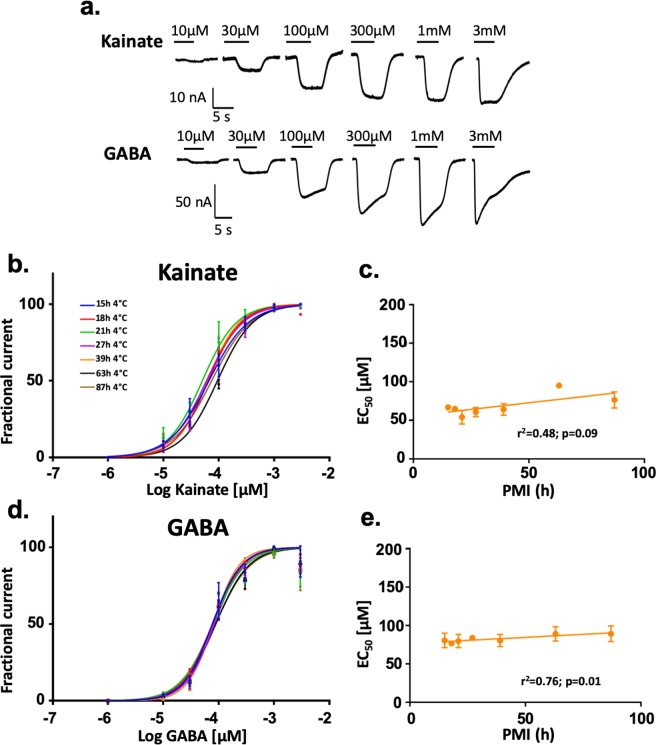
Human native synaptic receptors affinity is maintained for at least 87 h of PMI at morgue temperature. (**a**) Human native microtransplanted GABA and glutamate receptors responses at different PMI (15, 18, 21, 27, 39, 63, 87 h). (**b–d**) Concentration response curves for kainate and GABA (each point was calculated as the mean of 2–3 microtransplanted oocytes; see supplementary Table 5). Only oocytes with full dose responses and fits with p values <0.05 were included in the analysis. Curve fitting was done using the Hill equation I_current_ = 100/[1 + 10^((LogEC_50_-[agonist])*HillSlope)]. (**c–e)** EC_50_ calculated for each dose-response curve. All the groups were fit with linear regression.

An electrophysiological metric of global E/I ratio that is useful to compare brain regions/subjects in health and disease in large population cohorts should be simple to calculate, stable within each subject across experiments, and flexible enough to show pharmacological changes (Fig. 2). Because changes in excitatory plasticity are normally followed by homeostatic changes of inhibitory signaling we decided to allow pharmacological flexibility to the excitatory component of the E/I ratio by activating AMPARs with 100 µM kainate, which is near half of the apparent affinity (EC_50_) for kainate in human microtransplanted AMPARs (Figs. 4–5). Kainate is an agonist of AMPARs that keeps the channel in a non-desensitized state allowing consistent recordings of ion currents via AMPARs, produces less intra-subject variation compared to currents elicited by the combination of AMPA + cyclothiazide (CTZ), and due to the low expression of synaptic kainate-type receptors compared to AMPARs in synaptosomes, kainate-induced currents in microtransplanted oocytes reflect the activation of AMPARs^23^. For the estimation of the inhibitory component we used 1 mM GABA which is a saturating concentration of GABA_A_Rs. Thus, shifts of the E/I ratio reflect changes in the number and activity of excitatory or inhibitory receptors and/or small changes in AMPARs EC_50_ or large changes of GABA_A_Rs EC_50_. Figures 2 and 3 show kainate-induced AMPA currents and GABA currents recorded by two-electrode voltage clamp (TEVC) in rats and human microtransplanted native receptors between 20 and 32 hrs post-injection. Non-injected oocytes, as well as oocytes injected with total RNA extracted from rat synaptosomal preparations (supplementary Fig. 2b), were unresponsive to these agonists confirming that ion-currents elicited in injected oocytes were generated by microtransplanted synaptic receptors.

The temperature was critical for preservation of the amplitude of the currents in rats. No statistical differences with the control were found across PMIs at 2 °C, even 120 hrs after death (Fig. 2b–c; Kainate, *F* (5,106) = 2.035, p = 0.080; GABA, F (5,50) = 0.9342, p = 0.47 one-way ANOVA). In contrast, in samples stored at 21 °C ion currents gradually decreased with longer PMIs. After 6 hrs GABA currents were reduced by 57% and kainate-induced AMPA currents were reduced by 45%. No currents were observed in 21 °C/120 hrs samples. Remarkably, due to the uniform loss of activity of excitatory and inhibitory synaptic receptors the global E/I ratio was preserved at least up to 120 hrs at 2 °C, and up to 48 hrs at 21 °C (Fig. 2d; PMIs at 2 °C, *F* (5,50) = 1.077; p = 0.38 and PMIs at 21 °C; *F* (4,39) = 3.791, p = 0.01 using one-way ANOVA. 21 °C/48 hrs, p = 0.053; 21 °C/24 hrs, p = 0.94 using Dunnett’s multiple comparison). Moreover, the EC_50_ or slope (Hill coefficient) to kainate and GABA was not significantly affected by PMI or temperature (Fig. 4). Similar findings were observed in the DLPFC samples stored at 4 °C for different PMIs (Figs. 3 and 5), although the EC_50_ for kainate at 63 h was slightly higher than the rest. The biological activity of AMPA and GABA receptors showed a trend toward reduction by 30% after 87 hrs (Fig. 3b–c, AMPA *F* (6,33) = 0.2973, p = 0.93; GABA *F* (6,41) = 0.6263, p = 0.71) but the E/I ratio was preserved (Fig. 3d). These results strongly indicate that the variability of the E/I ratio is minimal compared to the measurements of excitatory or inhibitory components studied in isolation, and it is highly preserved across extremely large PMIs when the tissue is stored at morgue temperature.

It is important to note that our approach uses synaptic membranes that contain a mixed population of synapses, from different neuronal types; therefore, global changes of the E/I may be only able to detect severe alterations of the excitatory or inhibitory components, such as those observed in severe psychiatric and neurological disorders. State-dependent temporary changes of the E/I balance, or small alterations, could not be detected with this approach; Nevertheless, the global E/I also provides a way to normalize electrophysiological and pharmacological parameters of excitatory, or inhibitory, components using a within subject metric in future studies.

Our results provide strong support for the feasibility of electrophysiological and pharmacodynamic studies of the synaptic receptors responsible for the synaptic E/I ratio, using the invaluable tissue that is stored in brain banks over the world and in situations in which long PMIs are still unavoidable.

## Supplementary information

Supplementary information.
