# Supplementary material for: Preservation of global synaptic excitatory to inhibitory ratio during long postmortem intervals
Source: Sci Rep. 2020 May 25;10:8626. doi: 10.1038/s41598-020-65377-3 (PMC7248056; doi:10.1038/s41598-020-65377-3)
Supplement: Supplementary file 1 — Supplementary information. [file 41598_2020_65377_MOESM1_ESM.pdf]

Preservation of global synaptic excitatory to inhibitory ratio during long postmortem intervals

Pietro Scaduto[1,2], Adolfo Sequeira[3], Marquis P. Vawter[3], William Bunney[3], Agenor Limon[1*]

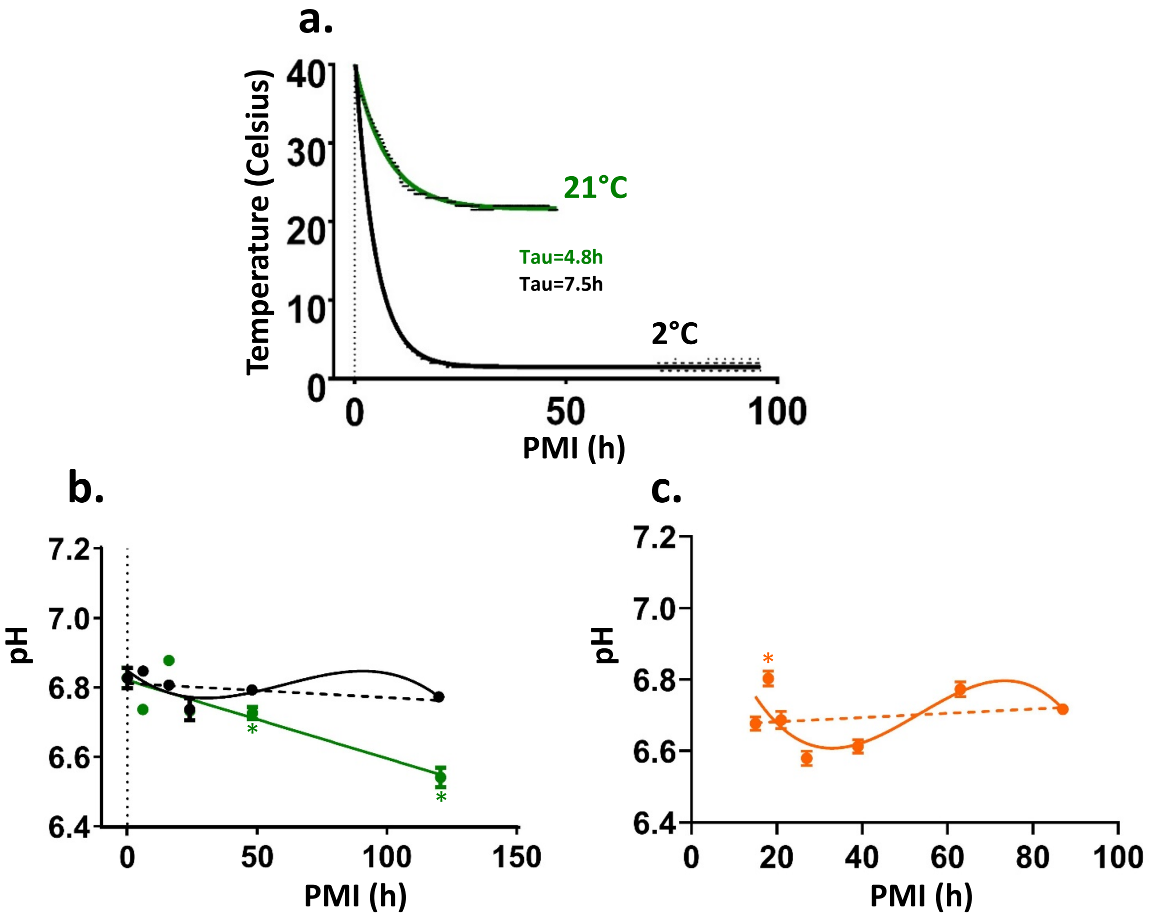

Supplementary figure 1. Temperature and pH. a. Temperature was monitored during the PMI. The curves were fitted with a one phase decay non-linear regression. **b.** The pH measurements show no differences across PMIs at 2°C (N=3) (fitted with third order polynomial equation). Instead, longer PMIs at 21°C (48h and 120h) show significative acidosis compared to the control at time 0 **c.** The pH of a human case kept at 4°C at different time intervals and fitted with third order polynomial equation. One-way ANOVA followed by Dunnett's multiple comparisons test; $p<0.05$ (N=3).

a.

	Sample	RIN
	Ctrl	7.7
4°C	6h	7.3
	16h	7.6
	24h	6.9
	48h	7.9
	120h	7
21°C	6h	7.4
	16h	6.7
	24h	7.7
	48h	7
	120h	2.5

b.

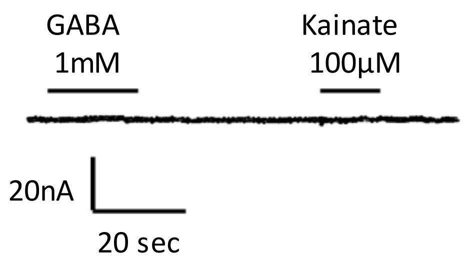

Supplementary figure 2. Synaptic RNA does not elicit *de novo* expression after 24-36 hrs after injection. a. Total isolated RNA showed an integrity number (RIN) with good quality across the experimental groups except in samples at 21°C for 120 h. **b.** Oocytes injected with total RNA from rat cortex, from the different experimental conditions, were recorded 24-36 h postinjection. No responses to GABA or kainate were observed, indicating electrophysiological currents shown after microtransplantation experiments were carried by synaptic receptors integrated in the oocytes membrane.

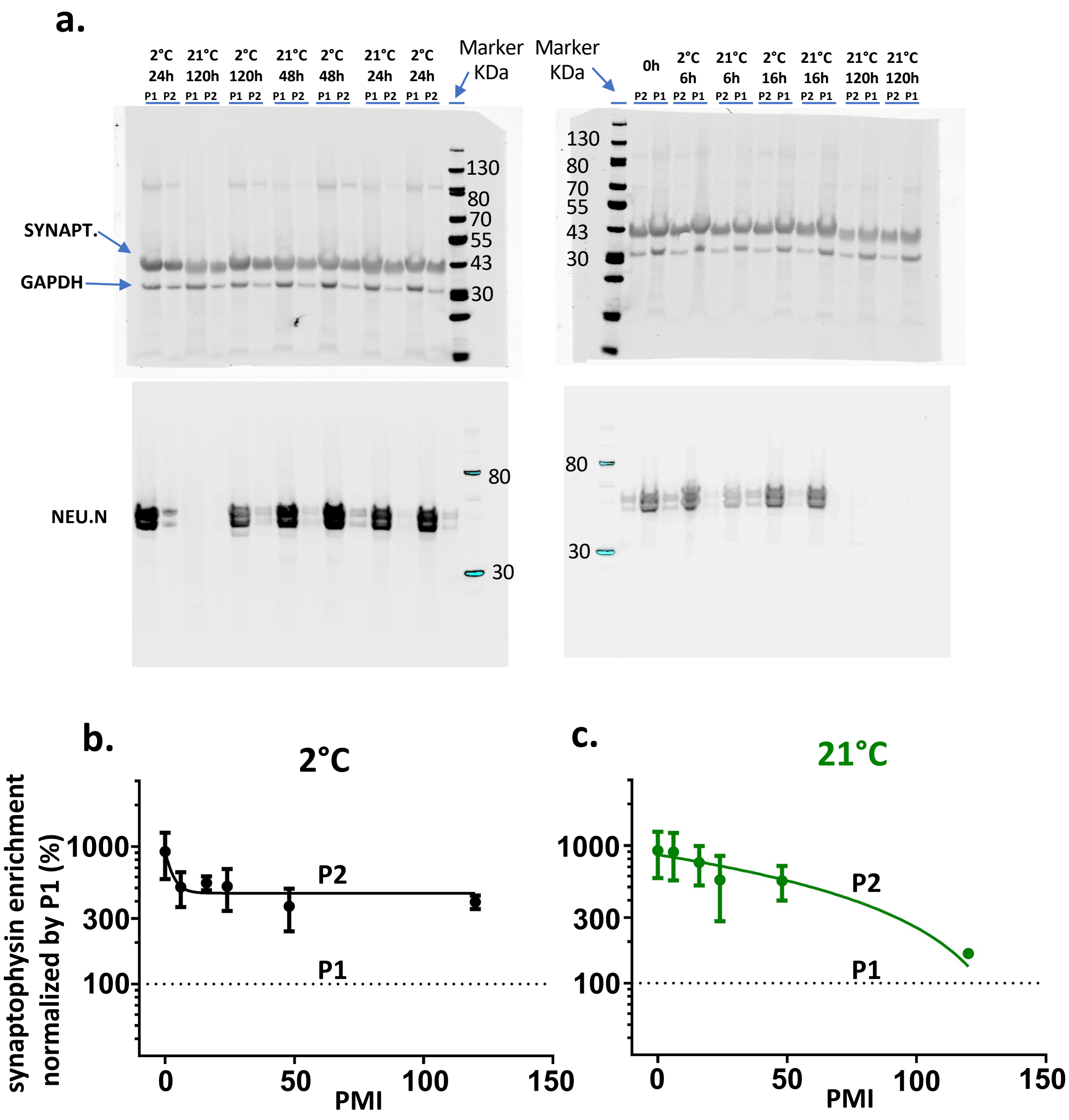

Supplementary figure 3. Synaptosome enrichment in P2 fraction. a. Two representative Western blots used for testing the quality of synaptosome preparation. The contrast was changed homogenously in the whole membrane to show the bands clearly. On top it is shown in gray tones the labeling by the secondary antibodies anti-mouse in red (top) and anti-rabbit in green (bottom). **b.-c.** Western blotting analysis of synaptophysin enrichment (calculated by the ratio of synaptophysin/Neu.N levels) in synaptosomes (P2) and nuclear fraction (P1, dotted line). The protein levels are expressed as percentage in a logarithmic scale. PMI time points at 2°C were described better with one phase decay exponential curve and at 21°C with linear regression equation. The enrichment of synaptosomes marker in P2 was significative in each experimental group except 120 h at 21°C, one-way ANOVA.

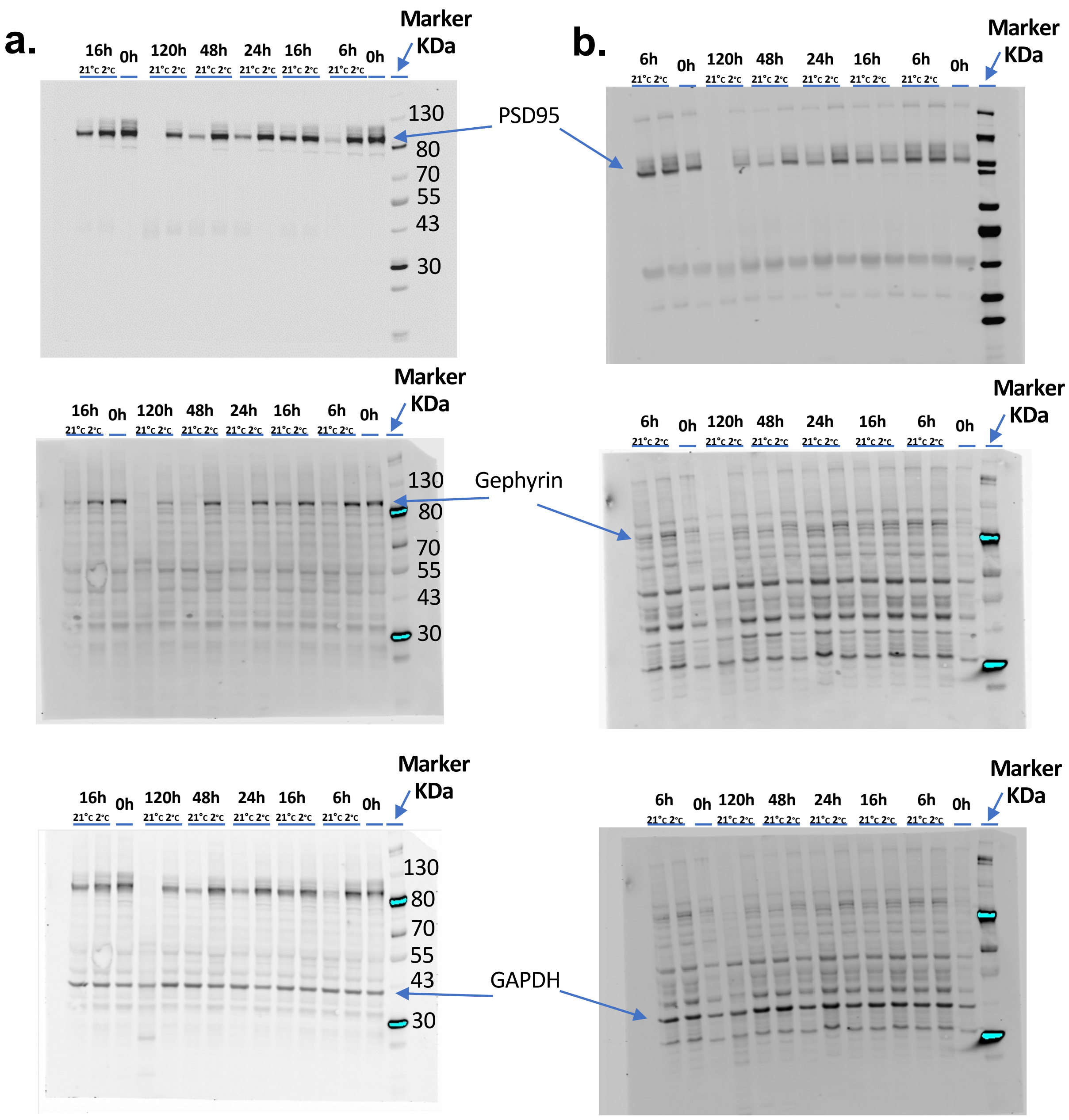

Supplementary Figure 4. a.-c. Full length western blotting gels. a.-b. Two of the membranes used to assess the levels of PSD-95, gephyrin, GAPDH (loading control). The contrast was modified homogenously in the whole membrane to ensure the best resolution depending on the intensity of the bands.

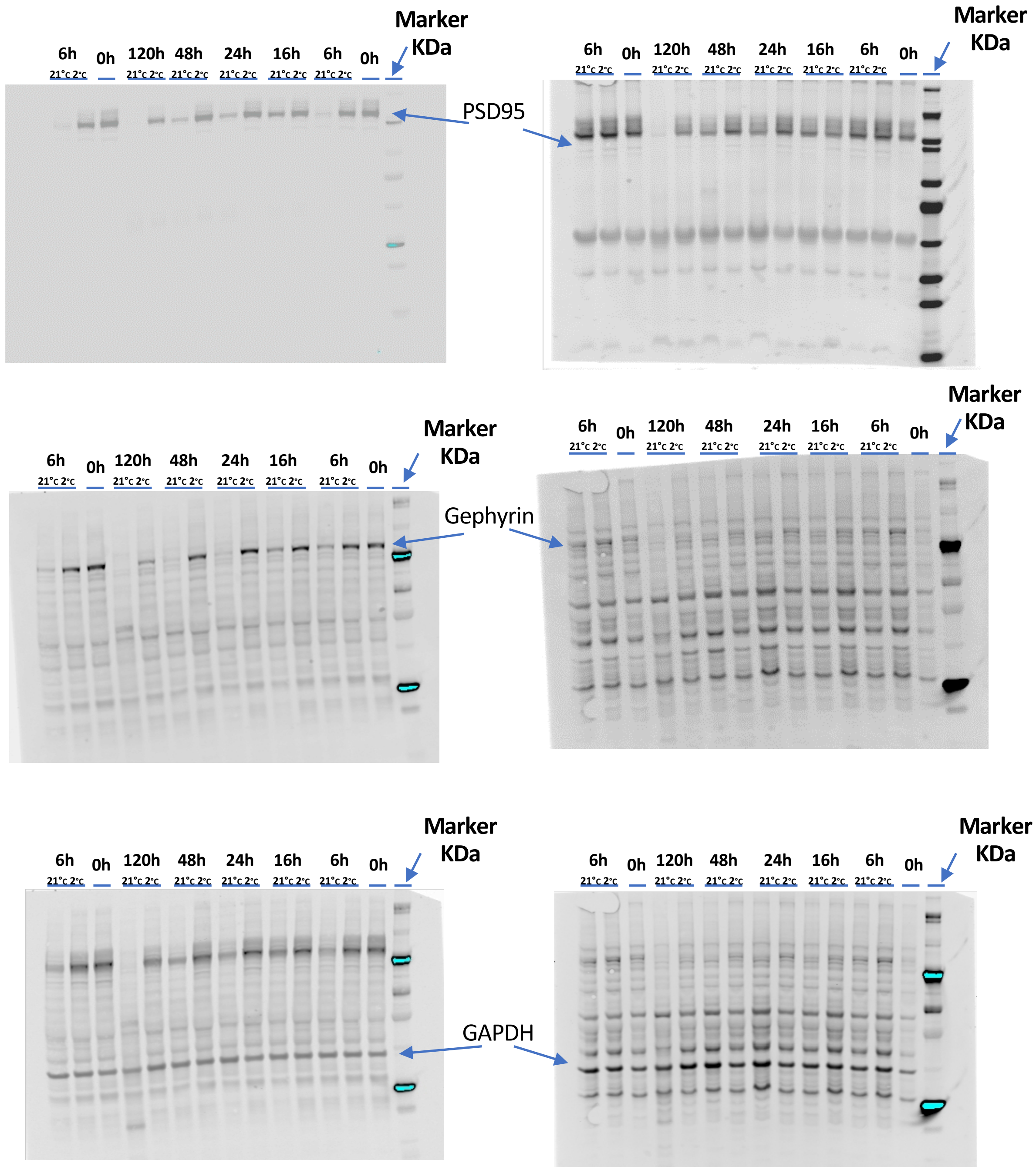

Supplementary Figure 5. a.-c. Full length western blotting gels. a.-b. Two other membranes used to assess the levels of PSD-95, gephyrin, GAPDH (loading control). The contrast was modified homogenously in the whole membrane to ensure the best resolution depending on the intensity of the bands.

Supplementary Table 1

**Figure 1**	Temperature (°C)	PMI (h)	N =gel bands	Temperature (°C)	PMI (h)	N = gel bands
		0	8			
	2	6	7	21	6	7
	2	16	5	21	16	5
	2	24	4	21	24	4
	2	48	4	21	48	4
	2	120	4	21	120	4

All the 4 membranes are displayed in supplementary figures 4 and 6.

Supplementary Table 2

Temperature (°C)	PMI (h)	N = oocytes	Temperature (°C)	PMI (h)	N = oocytes
	0	10/10/10			
2	6	10/10/10	21	6	11/10/10
2	16	9/9/9	21	16	8/7/7
2	24	9/9/9	21	24	8/8/8
2	48	9/9/9	21	48	8/8/8
2	120	8/8/8	21	120	1/0/0*

N = oocytes tested for GABA / kainate / ratio. * For 120 h/21°C we attempted to record 10 oocytes, only one had a very small response to GABA. The E/I ratio was calculated in oocytes where responses to GABA and kainate were clearly visible.

Supplementary Table 3

Temperature (°C)	PMI (h)	N = oocytes
4	15	12/11/11
4	18	6/6/6
4	21	6/6/6
4	27	6/5/5
4	39	6/4/4
4	63	6/3/3
4	87	6/5/5

N = oocytes tested for GABA / kainate / ratio. The E/I ratio was calculated in oocytes where responses to GABA and kainate were clearly visible.

Supplementary Table 4

Temperature (°C)	PMI (h)	N = oocytes	Temperature (°C)	PMI (h)	N = oocytes
	0	3/3			
2	6	3/3	21	6	3/3
2	16	3/3	21	16	3/3
2	24	3/3	21	24	3/2
2	48	3/3	21	48	2/2
2	120	3/3	21	120	0/0

N = oocytes tested for GABA / kainate. We tested 3 oocytes for each condition, only oocytes with full dose response and with a p value <0.05 for the fit of the Hill equation were used.

Supplementary Table 5

Temperature (°C)	PMI (h)	N = oocytes
4	15	3/3
4	18	3/2
4	21	3/3
4	27	3/3
4	39	3/2
4	63	3/2
4	87	3/2

N = oocytes tested for GABA / kainate. We tested 3 oocytes for each condition, only oocytes with full dose response and with a p value <0.05 for the fit of the Hill equation were used.
